# A Cofactor Regeneration System for 2‐Aminobutyric Acid Production Based on Combined Cross‐Linked Enzyme Aggregates: Utilizing His‐Tagged Enzymes With Low‐Concentration Calcium Ions as Precipitant

**DOI:** 10.1002/elsc.70013

**Published:** 2025-02-28

**Authors:** Jingran Liu, Ren Li, Jincheng Miao, Hongxu Sun, Qiwei Chen, Haiyan Song, Hui Peng, Yanhong Chang, Hui Luo

**Affiliations:** ^1^ Department of Environmental Science and Engineering University of Science and Technology Beijing Beijing China; ^2^ Department of Biological Science and Engineering University of Science and Technology Beijing Beijing China; ^3^ Beijing Key Laboratory of Resource‐Oriented Treatment of Industrial Pollutants Beijing China

**Keywords:** 2‐aminobutyric acid, calcium ions, cofactor regeneration, combi‐CLEAs

## Abstract

Combined cross‐linked enzyme aggregates (combi‐CLEAs) represent a promising carrier‐free immobilized enzyme technology. This study describes the preparation of combi‐CLEAs comprising leucine dehydrogenase (LeuDH) and formate dehydrogenase (FDH) for the regeneration of cofactor nicotinamide adenine dinucleotide necessary for 2‐aminobutyric acid production. Different from traditional methods using ammonium sulfate or organic reagents as precipitant, this work utilized low concentrations of calcium ions to purify and precipitate the histidine‐tagged enzymes. We developed a simple and environmentally friendly protocol for combi‐CLEAs formation, involving precipitation with 10 mM calcium ions at an enzyme activity ratio of 1:2 for LeuDH and FDH, respectively, followed by cross‐linking with 0.15% (w/v) glutaraldehyde at 20°C for 2 h at pH 7.5. The optimal catalytic reaction temperature and pH value for the combi‐CLEAs were determined to be a temperature of 37°C and a pH of 7.5. The combi‐CLEAs demonstrated enhanced thermal and pH tolerance compared to the free enzyme mixture. Moreover, the combi‐CLEAs showed good operational stability, retaining 40% of its initial activity after seven cycles of reuse. These findings suggest that the combi‐CLEAs of LeuDH and FDH are an efficient and cost‐effective option for 2‐aminobutyric acid production.

**Abbreviations**
Combi‐CLEAs: combined cross‐linked enzyme aggregates
FDH: formate dehydrogenase
His‐tag: histidine tag
LeuDH: leucine dehydrogenase

## 1 Introduction

Over 80% of oxidoreductases in nature require nicotinamide adenine dinucleotide (NAD^+^) or its phosphorylated form (NADPH) as cofactors for transferring electrons and protons in redox reactions. The stoichiometric use of NAD(P)H in large‐scale synthesis is not economically feasible given their extremely high cost. Thus, in situ regeneration of nicotinamide cofactors is necessary for industrial‐scale biosynthesis [1]. This not only saves costs but also reduces resource consumption and is environmentally friendly.

**Summary**

- Our research introduces an innovative method for preparing combined cross‐linked enzyme aggregates (combi‐CLEAs) of leucine dehydrogenase (LeuDH) and formate dehydrogenase (FDH) to regenerate nicotinamide adenine dinucleotide in 2‐aminobutyric acid production.
- Utilizing low concentrations of calcium ions as a precipitant for histidine (His)‐tagged enzymes, we provide a simple, cost‐effective, and environmentally friendly alternative to conventional enzyme immobilization methods that rely on high amounts of ammonium sulfate or organic solvents.
- The combi‐CLEAs developed through this method display enhanced thermal and pH stability, as well as superior operational stability, which are essential for sustainable industrial bioprocessing.
- This approach is particularly advantageous for the production of 2‐aminobutyric acid, offering a promising strategy for the use of recombinant His‐tagged enzymes in biocatalysis.
- Moreover, the use of calcium ions as a precipitant may pave the way for new enzyme purification techniques and biocatalyst development.

**FIGURE 1:**
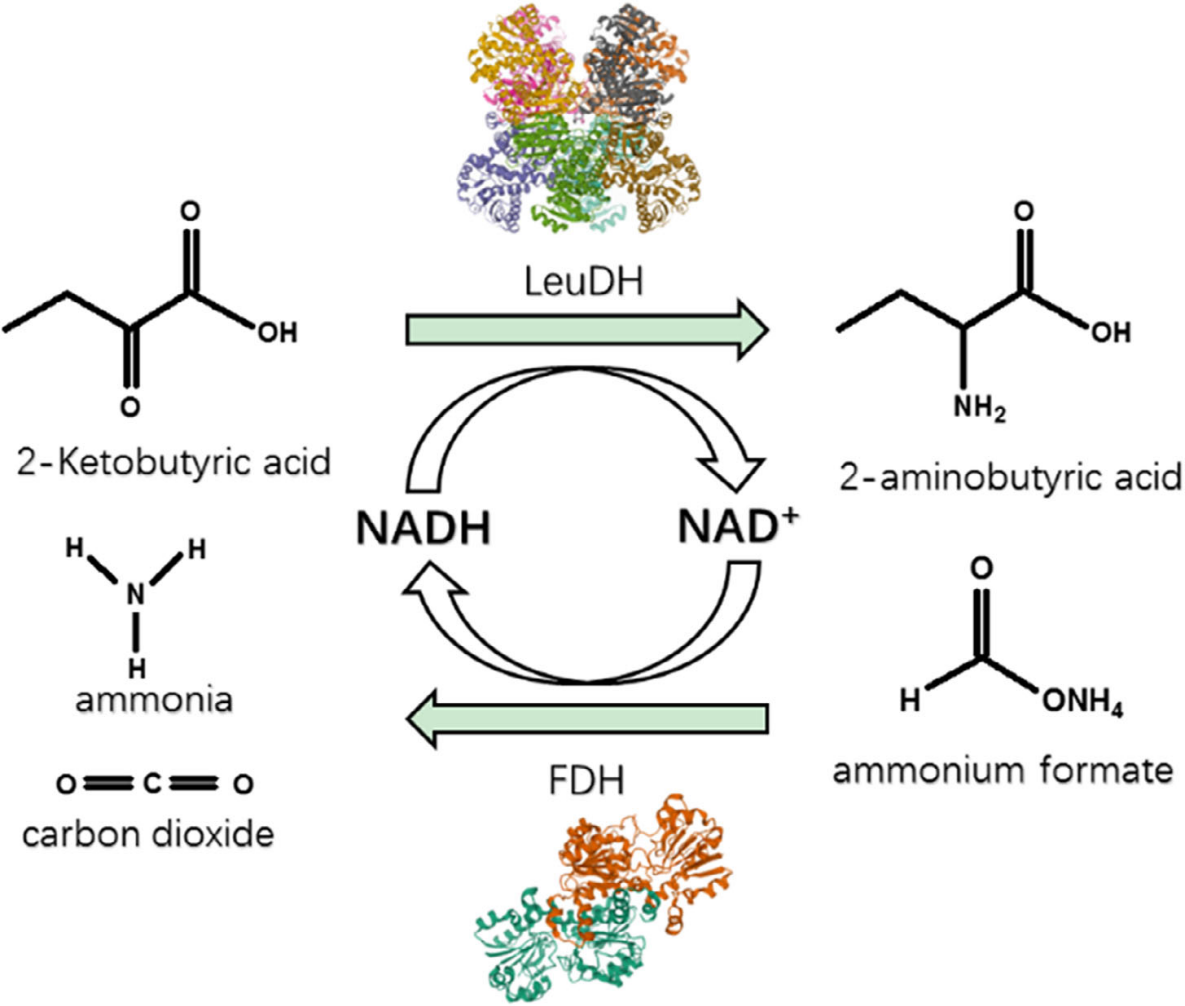
The biosynthesis of 2‐aminobutyric acid through the dehydrogenation reaction catalyzed by LeuDH and the cofactor NADH cycle regeneration achieved after introducing FDH.

2‐Aminobutyric acid, an unnatural amino acid, which promotes the metabolism of human brain cells and reduces cerebral blood pressure, can be used as a key intermediate for the synthesis of several important drugs, such as levetiracetam, ethambutol, and brivaracetam [2, 3]. Currently, the preparation of 2‐aminobutyric acid is mainly achieved by chemical synthesis or enzymatic conversion. However, the chemical synthesis method presents significant drawbacks, including poor selectivity, harsh reaction conditions, and the production of various byproducts. In light of growing concerns about serious concerns about climate change and environmental issues, enzymatic synthesis of 2‐aminobutyric acid has garnered increasing attention [4]. Among enzymatic conversion methods, 2‐aminobutyric acid can be synthesized by reducing 2‐ketobutyric acids using leucine dehydrogenase (LeuDH, EC 1.4.1.9). In this process, cofactor NADH needs to be regenerated that can be addressed by catalyzing ammonium formate with formate dehydrogenase (FDH, EC 1.2.1.2) (Figure 1). This approach is commonly used for the NADH regeneration in biocatalysis and biotransformation [5]. To enhance the efficiency of the cofactor regeneration system, strategies such as co‐immobilizing the related enzymes and improving their tolerance to extreme conditions have been employed. Xu et al. [6] co‐immobilized LeuDH and FDH using epoxy resins or amino resins as carriers, which facilitated the hydrogenation reaction and increased the conversion rate of 2‐ketobutyric acids. As a result, the yield of the product 2‐aminobutyric acid could exceed 90% [6].

In industrial processes, free enzymes often face significant challenges due to their inherent instability. The harsh conditions prevalent in these settings, such as extreme temperatures, pressures, and pH levels, can lead to the denaturation of free enzymes, thereby reducing their catalytic efficiency and shortening their operational lifespan [7, 8, 9]. This instability not only hampers the economic viability of enzyme‐catalyzed reactions but also complicates the separation of products from the reaction mixture, adding to the overall cost and complexity of the process. The immobilization of enzymes offers a promising solution to these issues [10, 11]. By attaching enzymes to a solid support, their stability can be significantly enhanced, allowing them to maintain their activity over a broader range of conditions. Moreover, immobilized enzymes can be easily separated from the reaction mixture, facilitating product purification and enabling the enzymes to be reused multiple times, which is a significant advantage in terms of cost‐effectiveness and sustainability [12].

Immobilized enzymes are physically or chemically confined or localized within a certain region or space, retaining their catalytic activity over numerous reaction cycles [7, 13]. Immobilized enzyme technology can be categorized into two main approaches: immobilization with a carrier and immobilization without a carrier [9, 14, 15]. When enzymes are bound to or encapsulation within a carrier, there is a potential dilution of catalytic activity and reduced productivities (kilograms of product per kilogram of enzyme) owing to the significant proportion (90%–99% of the total mass) of noncatalytic material introduced by the carrier itself. Moreover, carriers used for immobilization are often costly, which can be a limiting factor in the economic viability of enzyme immobilization processes [16].

In recent years, the immobilization of enzymes as cross‐linked enzyme aggregates (CLEAs) has attracted considerable interest, especially due to their potential applications in industrial biocatalysis [17, 18]. The advent of combined cross‐linked enzyme aggregates (combi‐CLEAs) has introduced an efficient, carrier‐free immobilization technique that is particularly well suited for multi‐enzyme co‐immobilization and cascade catalytic reactions. Combi‐CLEAs are typically prepared through a process involving the aggregation of several enzymes, induced by salt or solvent, followed by cross‐linking with bifunctional agents such as glutaraldehyde or polymers, resulting in a stable all‐protein precipitate [19, 20]. To date, various combi‐CLEAs have been reported [21, 22, 23]. For the preparation of combi‐CLEAs, neutral salt ammonium sulfate is commonly used as a precipitant [24], whereas some researchers employ organic solvents as precipitants [25, 26, 27, 28, 29]. The addition of a high concentration of neutral salt to a protein solution can disrupt the protein's hydration layer and ionosphere, neutralizing the surface charge and prompting the proteins to aggregate and precipitate. Similarly, the introduction of organic solvents to a protein solution reduces its electrolysis constant and disrupts the hydration layer on the protein surface, causing the free enzyme molecules in the solution to precipitate out [30, 31]. However, these conventional precipitation methods for preparing combi‐CLEAs often require high doses and concentrations of ammonium sulfate or organic solvents. These methods can lead to the discharge of excessive ammonia nitrogen or organic reagents in wastewater during industrial production, thereby complicating wastewater treatment. Moreover, these methods often prioritize protein concentration over purification. Therefore, there is an urgent need to develop a precipitation method that uses low concentrations of salts and exhibits high selectivity for target protein precipitation.

In the field of biotechnology, protein purification remains a costly endeavor. With the increasing development of recombinant proteins, there is a growing trend toward utilizing immobilized metal ion affinity chromatography for their purification, particularly through the use of histidine tag (His‐tag). Our research group has recently developed a novel method known as cation affinity purification (CAP), which offers an efficient and cost‐effective approach for purifying multi‐subunit recombinant proteins that incorporate His‐tags [32]. This method is characterized by its minimal salt usage and results in the purified protein being obtained in a precipitated state. This characteristic is particularly advantageous for the subsequent development of CLEAs.

In this work, we compared the conventional precipitant, ammonium sulfate and organic reagents (ethanol and acetone), with novel method (CAP) using low‐concentration calcium or magnesium salts as precipitant for the precipitation of the recombinant His‐tagged protein. The mechanism of low‐concentration calcium ions purifying and precipitating the his‐tagged proteins was speculated. The combi‐CLEAs of LeuDH and FDH were prepared by cross‐linking using glutaraldehyde after coprecipitating with 10 mM calcium ions, and the preparation parameters and catalytic efficiency of the combi‐CLEAs were further explored.

## 2 Materials and Methods

### 2.1 Materials

*Escherichia coli* strain BL21 (DE3) (Tiangen, Beijing, China) was used as the host for the recombinant plasmids pET‐LeuDH and pET‐FDH, which were derived from pET‐28a. These plasmids were employed for the expression of LeuDH and FDH with a six‐His‐tag fused to their N‐terminus (the gene sequences are shown in the Supplementary Material). Ammonium formate, 2‐aminobutyric acid, and 2‐ketobutyric acid were purchased from Macklin (Shanghai, China). BCA Protein Assay Kit (Solarbio, Beijing, China) was used to determine protein concentration. All other chemicals used were of analytical grade.

### 2.2 Plasmid Construction and Protein Expression

The plasmids pET‐LeuDH and pET‐FDH were constructed using the Golden Gate cloning method [33], and they were then transformed into *E. coli* BL21 (DE3). The resulting recombinant strains were cultured in 3 mL of Luria–Bertani (LB) medium (NaCl 10 g/L, peptone 10 g/L, yeast extract 5 g/L, and kanamycin 50 µg/mL) at 37°C and 200 rpm for 12 h. The culture was then transferred to 50 mL of lactose auto‐induction medium (peptone 10 g/L, yeast extract 5 g/L, Na_2_HPO_4_ 8.95 g/L, KH_2_PO_4_ 3.4 g/L, NH_4_Cl 2.67 g/L, Na_2_SO_4_ 0.7 g/L, MgSO_4_ 0.24 g/L, glycerin 5 g/L, glucose 0.5 g/L, lactose 2 g/L, and kanamycin 50 µg/mL) with 2.5% inoculum at 28°C and 200 rpm for 24 h. The harvested cells were suspended in deionized water, and cell extracts were prepared by sonication and centrifuged at 4°C.

### 2.3 Precipitation of Enzymes

The mixed solution of LeuDH and FDH, prepared at an enzymatic activity ratio of 1:1, was subjected to precipitation using varying concentrations of ethanol (100%, 70%, and 40% v/v), acetone (100%, 70%, and 40% v/v), and saturated ammonium sulfate (100%, 70%, and 40% v/v). Concurrently, we compared these traditional methods with the use of CaCl_2_ or MgCl_2_ as precipitants, with final concentrations of 5, 10, 25, 50, and 100 mM. The precipitation process was conducted by adding 9 mL of the respective precipitant solutions to 1 mL of the mixed enzyme solution at 4°C for 60 min, followed by centrifugation at 5500 rpm for 15 min. The supernatant was collected, and the precipitated proteins were redissolved in an equal volume of buffer (0.1 mM sodium phosphate buffer, pH 7.5). The activity recovery percentage of LeuDH and FDH in both supernatant and the precipitate was determined using the following Equation (1):

(1) $$\begin{array}{c} \mathrm{Activity}\;\mathrm{recovery}\;\left(\%\right)=\frac{\mathrm{Total}\;\mathrm{activity}\;\mathrm{of}\;\mathrm{each}\;\mathrm{enzyme}\;\mathrm{in}\;\mathrm{supernatant}\;\mathrm{or}\;\mathrm{redissolved}\;\mathrm{precipitation}\;\left(U/\mathrm{mL}\right)}{\mathrm{Total}\;\mathrm{inital}\;\mathrm{activity}\;\mathrm{of}\;\mathrm{each}\;\mathrm{free}\;\mathrm{enzyme}\;\left(U/\mathrm{mL}\right)}\times 100\% \end{array}$$

Protein concentrations of redissolved precipitate were measured using the BCA Protein Assay Kit, according to the manufacturer's instructions. The specific activity of LeuDH or FDH in the precipitate was assayed by the following Equation (2):

(2) $$\mathrm{Specific}\;\mathrm{activity}\;\left(U/\mathrm{mg}\right)=\frac{\mathrm{Total}\;\mathrm{activity}\;\mathrm{of}\;\mathrm{each}\;\mathrm{enzyme}\;\mathrm{in}\;\mathrm{redissolved}\;\mathrm{precipitation}\;\left(U/\mathrm{mL}\right)}{\mathrm{Protein}\;\mathrm{concentrations}\;\mathrm{of}\;\mathrm{each}\;\mathrm{enzyme}\;\mathrm{in}\;\mathrm{redissolved}\;\mathrm{precipitation}\;\left(\mathrm{mg}/\mathrm{mL}\right)}$$

### 2.4 Preparation of Combi‐CLEAs

For the preparation of combi‐CLEAs, a 16 mL solution containing LeuDH and FDH at their determined activity ratio was subjected to precipitation by the addition of CaCl_2_ solution to achieve a final concentration of 10 mM at 4°C for 60 min. The mixture was then centrifuged at 5500 rpm for 5 min, and the supernatant was carefully discarded. Subsequent steps involved the drop‐wise addition of glutaraldehyde at various final concentrations (0.10–0.45% w/v) to the precipitation, followed by incubation at temperatures ranging from 15°C to 37°C and pH values adjusted between 7.0 and 9.0 (using sodium phosphate buffer pH for 6.0–8.0, and ammonia–ammonium chloride buffer for pH 8.5–9.0) for different time intervals (0.5–7.0 h). After the incubation, the mixtures were centrifuged at 5500 rpm for 15 min to obtain the supernatants and precipitates. The supernatant was discarded, and the pellet was washed with 0.1 M sodium phosphate buffer (pH 7.5) until no enzyme activity was detectable in the supernatant. The resulting combi‐CLEAs were then stored in 0.1 M sodium phosphate buffer (pH 7.5) at 4°C for further use.

### 2.5 Determination of Enzyme Activity

The enzyme activity of LeuDH was assayed toward 4.5 mM 2‐ketobutyric acid (dissolving in 900 mM pH 9.5 NH_3_‐NH_4_Cl buffer, containing 0.204 mM NADH) at 30°C using an EnSpire 2300 Multi‐Function Plate Reader (PerkinElmer, Waltham, MA, USA), and the enzyme activity was calculated by measuring the change in NADH concentration at 340 nm over a period of 3 min. One unit (U) of LeuDH activity is defined as the amount of enzyme required to consume 1 µmol of NADH per minute.

Similarly, the enzyme activity of FDH was determined toward 167 mM ammonium formate and 1.67 mM NAD^+^ (dissolving in 100 mM pH 7.5 sodium phosphate buffer) at 30°C using the same plate reader, and the enzyme activity was calculated by measuring the NADH concentration change at 340 nm over 3 min. One unit (U) of FDH activity is defined as the amount of enzyme required to produce 1 µmol of NADH per minute.

The activity of combi‐CLEAs and the free enzyme mixture of LeuDH and FDH was evaluated using 2‐ketobutyric acids as the substrate. The reaction product, 2‐aminobutyric acid, was analyzed using high‐performance liquid chromatography (HPLC) with Phenomenex Luna C‐18 column (4.6 × 150 mm). The mobile phase consisted of 15% methanol, and the flow rate was set at 1 mL/min, with a detection wavelength of 210 nm. The standard assay conditions were as follows: a total volume of 5 mL containing 50 mM 2‐ketobutyric acids, 50 mM ammonium formate, 0.5 mM NAD^+^, 0.1 M phosphoric acid buffer (pH 7.5), and an appropriate amount of combi‐CLEAs or free enzymes mixture, incubated at 200 rpm at 37°C for 15 min. A unit (U) of combi‐CLEAs (or free enzymes mixture of LeuDH and FDH) activity is defined as 1 µmol of 2‐aminobutyric acid produced per minute and per milligram of wet immobilisate (or per milliliter of mixed LeuDH and FDH solution). All experiments were performed in triplicate.

### 2.6 Characterization of Combi‐CLEAs

The influence of reaction pH on the activities of combi‐CLEAs and the free enzyme mixture of LeuDH and FDH (at an enzymatic activity ratio of 1:2) was evaluated under standard assay conditions, with the exception of using buffers with different pH values. The pH values tested were 6.0, 6.5, 7.0, 7.5, 8.0, 8.5, and 9.0, using sodium phosphate buffer for pH 6.0–8.0, and ammonia–ammonium chloride buffer for pH 8.5–9.0. Additionally, the effect of reaction temperature on the activities was assessed as mentioned above at the different temperatures of 17°C, 27°C, 37°C, 47°C, and 57°C.

The thermal stability of combi‐CLEAs and the free enzymes mixture was investigated by incubating them in 0.1 M sodium phosphate buffer (pH 7.5) without substrate at 45°C for various durations. Samples of catalysts were taken at different time intervals to determine the residual activity.

The pH tolerance of combi‐CLEAs and the free enzyme mixture was evaluated at 25°C for various durations in 0.1 M sodium phosphate buffer at pH 6.0 or 0.1 M ammonia–ammonium chloride buffer at pH 10.0, without substrate. The activity of catalyst samples was measured using the method as described above.

The reusability of enzymes in combi‐CLEAs was investigated under standard assay conditions. After each catalytic reaction cycle, the combi‐CLEAs were separated by centrifugation, washed twice with a 0.1 M sodium phosphate buffer (pH 7.5), and then resuspended in fresh substrate. The activity of the combi‐CLEAs from the first cycle was set as the 100% activity control.

The surface morphology of combi‐CLEAs in a freeze‐dried form was analyzed using a scanning electron microscope (SU8010, Hitachi, Tokyo, Japan). The combi‐CLEAs samples were freeze dried, fixed onto an aluminum stub, and coated with a thin film of platinum. The surface properties and morphology of the combi‐CLEAs were studied.

Confocal laser scanning microscopy (CLSM) was used to investigate the distribution of LeuDH and FDH within the combi‐CLEAs. Prior to being observed, the enzyme solutions of LeuDH and FDH were mixed with fluorescein isothiocyanate (FITC) (1 mg/mL) and Rhodamine B (RhoB) (1 mg/mL), respectively, for 24 h, and then the two enzymes were prepared as combi‐CLEAs. CLSM observations were performed with a confocal fluorescence microscope (FV‐1200, Olympus, Tokyo, Japan). The samples were excited at 495 and 570 nm.

The kinetic parameters *K*_m_ and *V*_max_ of free enzyme mixture of LeuDH and FDH and combi‐CLEAs were determined by measuring reaction rates at varying concentrations of 2‐ketobutyric acid (10–50 mM). Data were fitted to the Michaelis–Menten equation, and the Lineweaver–Burk plot was used for linear regression analysis.

## 3 Results and Discussion

### 3.1 Selection of Precipitant

**FIGURE 2:**
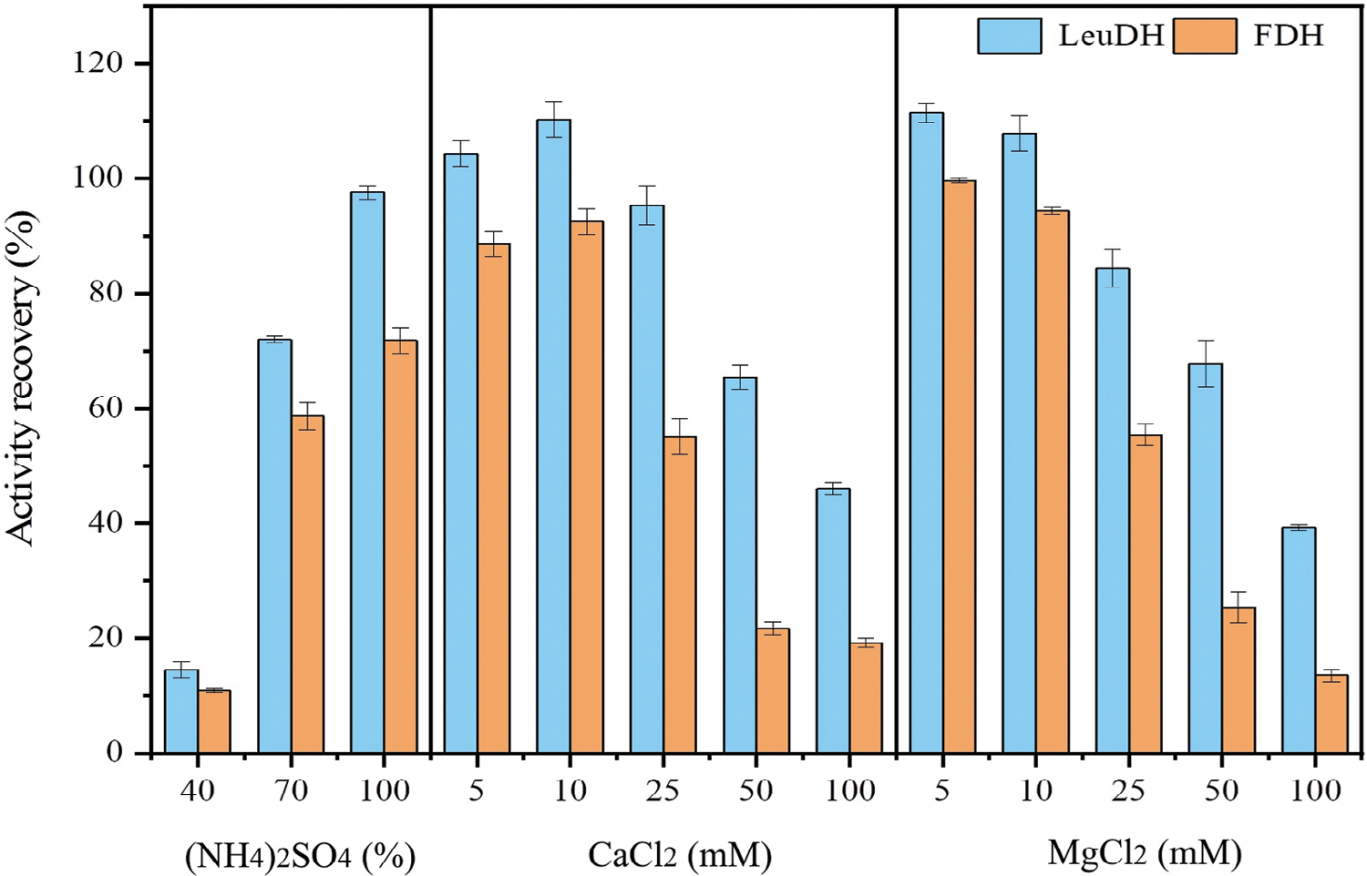
Effect of different precipitants at different concentrations on the activity recovery of LeuDH and FDH in coprecipitation. The molar concentrations of 40%, 70%, and 100% (v/v) saturated ammonium sulfate are 2.32, 4.07, and 5.81 M, respectively. The experiments were conducted in triplicate, and the error bars represent the standard deviation of the mean activity values.

Different concentrations of precipitants, including ethanol, acetone, and ammonium sulfate, were tested for coprecipitation for the crude enzyme solution of LeuDH and FDH (enzyme activity ratio was 1:1), and the results were compared with those obtained using calcium and magnesium salts. The findings indicated that when ethanol or acetone was added, the enzyme activities in both the supernatant and precipitation were very low (Table 1), suggesting that the inactivation of the enzymes during coprecipitation may be attributed to their poor tolerance to ethanol or acetone. The activity recovery in precipitation using different concentrations of ammonium sulfate, calcium, or magnesium ions as precipitants is shown in Figure 2. It is evident that the activity recovery of LeuDH and FDH in the precipitate was significantly higher compared to that observed with ethanol and acetone (Table 1). Notably, when comparing with the highest recovery achieved with ammonium sulfate (100% v/v 5.81 M), the novel method employing lower concentration (5 or 10 mM) calcium or magnesium ions as precipitants yielded superior activity recovery. This suggests that the novel method is advantageous not only for enhancing activity recovery but also for minimizing the amount of precipitant used. It was also observed that when the concentrations of calcium and magnesium ions exceeded 10 mM, the activity recovery decreased rapidly. Figure 2 reveals that the enzyme activity recovery of LeuDH and FDH was optimal at a concentration of 10 mM calcium ions. Therefore, 10 mM calcium ions were selected as the precipitant for the preparation of combi‐CLEAs.

**TABLE 1:** Activity recovery in supernatant and precipitation with ethanol or acetone as precipitant.

		LeuDH		FDH	
Precipitant	Concentration/% (v/v)	Activity recovery in supernatant/%	Activity recovery in precipitation/%	Activity recovery in supernatant/%	Activity recovery in precipitation/%
Ethanol	40	0.571 ± 0.1	1.000 ± 0.2	0.132 ± 0.1	0.132 ± 0.1
	70	1.000 ± 0.1	0.286 ± 0.1	0.132 ± 0.1	0.132 ± 0.1
	100	2.714 ± 0.2	5.429 ± 0.2	0.526 ± 0.1	0.184 ± 0.2
Acetone	40	5.429 ± 0.6	1.857 ± 0.2	0.132 ± 0.1	ND
	70	7.000 ± 0.2	2.714 ± 0.1	0.132 ± 0.2	ND
	100	7.000 ± 0.5	2.286 ± 0.1	0.132 ± 0.1	ND

*Note:* The experiments were conducted in triplicate, and the error represents the percentage error in each set of readings. ND indicates no detectable activity.

In order to compare the effect of traditional precipitant ammonium sulfate with low‐concentration calcium and magnesium ions on the protein purification rate, the specific activities of LeuDH and FDH in coprecipitation were determined (Table 2). It was obvious that low‐concentration calcium or magnesium ions yielded higher specific enzyme activity compared to ammonium sulfate. This observation led us to hypothesize about the mechanisms underlying the superior performance of calcium or magnesium ions in the precipitation and purification of LeuDH and FDH.

**TABLE 2:** Effect of different precipitants at different concentrations on the specific enzyme activity of LeuDH and FDH in coprecipitation.

		Specific enzyme activity (U/mg)	
Precipitant	Concentration	LeuDH	FDH
(NH_4_)_2_SO_4_	40% (v/v)	0.217	0.181
	70% (v/v)	0.237	0.189
	100% (v/v)	0.279	0.220
CaCl_2_	5 mM	0.449	0.333
	10 mM	0.455	0.308
	25 mM	0.371	0.286
	50 mM	0.321	0.240
	100 mM	0.379	0.260
MgCl_2_	5 mM	0.442	0.308
	10 mM	0.424	0.306
	25 mM	0.372	0.316
	50 mM	0.438	0.300
	100 mM	0.353	0.196

*Note:* The molar concentrations of 40%, 70%, and 100% (v/v) saturated ammonium sulfate are 2.32, 4.07, and 5.81 M.

It is well known that a six‐His‐tag is usually used as an affinity purification tag, which is typically introduced at the N‐ or C‐terminus of the target proteins. In contrast, host cell proteins lacking a His‐tag are present as impurities in the crude enzyme solution. We propose that the aggregation of LeuDH and FDH is facilitated by the interaction between calcium or magnesium ions with the His‐tag on these enzymes, whereas impurity proteins lacking His‐tags are less likely to form precipitates [32]. This suggests that calcium or magnesium ions can serve dual roles as precipitants and as agents for purifying target proteins.

**FIGURE 3:**
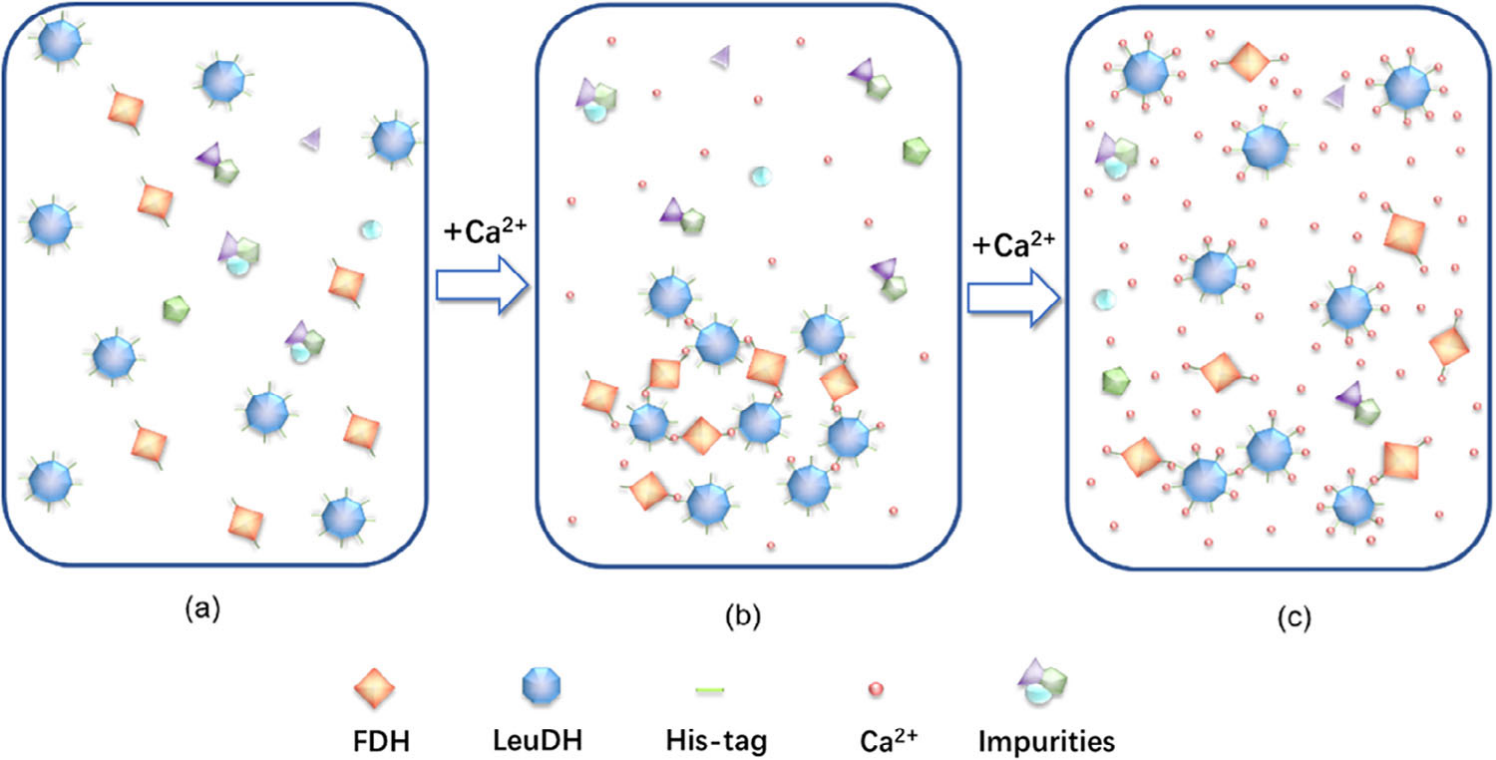
Schematic diagram of the interaction between calcium ions and His‐tag.

Upon increasing the concentration of calcium ions (calcium ions as an example), we observed a gradual decline in the activity recovery of LeuDH and FDH in coprecipitation (Figure 2). This decrease may be due to the competitive binding of calcium ions at higher concentrations. Since the crude enzyme extract was prepared with deionized water in this work, resulting in a low ion content, and considering the abundance of consecutive histidine imidazole groups in the recombinant proteins (Figure 3a), we can infer the following mechanism: At low calcium ion concentrations (which could precipitate LeuDH and FDH), most ions likely contribute to the compression of the protein's electrical double layers, allowing His‐tags to be exposed and brought closer together. The imidazole groups can then form complexes with calcium ions in a 1:x ratio (one calcium ion interacting with two or more imidazole groups). In this scenario, calcium ions act as physical crosslinkers, prompting the formation of supramolecular aggregates of multimeric LeuDH and FDH (Figure 3b). However, as cation concentration increases, more calcium ions participate in and tend to form 1:1 complexes with imidazole groups, reducing the cross‐linking degree of LeuDH and FDH (Figure 3c), which in turn causes the enzyme aggregates to gradually dissolve and revert to a soluble state [32].

Moreover, when calcium or magnesium ions were used as precipitant, it was noted that the activity recovery of LeuDH in coprecipitation was higher than that of FDH (Figure 2). This disparity may be attributed to the fact that LeuDH is a homologous octamer, whereas FDH is a homodimer. The formation of precipitation is likely a result of the interaction of two or more imidazole groups on His‐tags of different enzyme molecules with calcium or magnesium ions, leading to the aggregation of the target proteins. Therefore, proteins with a higher number of tags are more likely to transition from solution to precipitation at low concentrations of calcium or magnesium ions.

To achieve a moderate enzyme recovery rate and purity, conventional methods using ammonium sulfate as a precipitant typically require a 70% saturation level, equivalent to a concentration of 4.07 M. This concentration is 2–3 orders of magnitude higher than the low concentrations of calcium or magnesium ions (5 or 10 mM) used as precipitants in our study. Consequently, the cost of the precipitant for enzyme precipitation in our method is only 1%–2% of that associated with conventional methods. Furthermore, considering that high concentrations of ammonium sulfate can lead to nitrogen pollution in wastewater, the use of low‐concentration calcium or magnesium ions for enzyme precipitation offers significant environmental benefits.

### 3.2 Optimization of Cross‐Linking Parameters

#### 3.2.1 Effect of Activity Ratio of LeuDH and FDH

**FIGURE 4:**
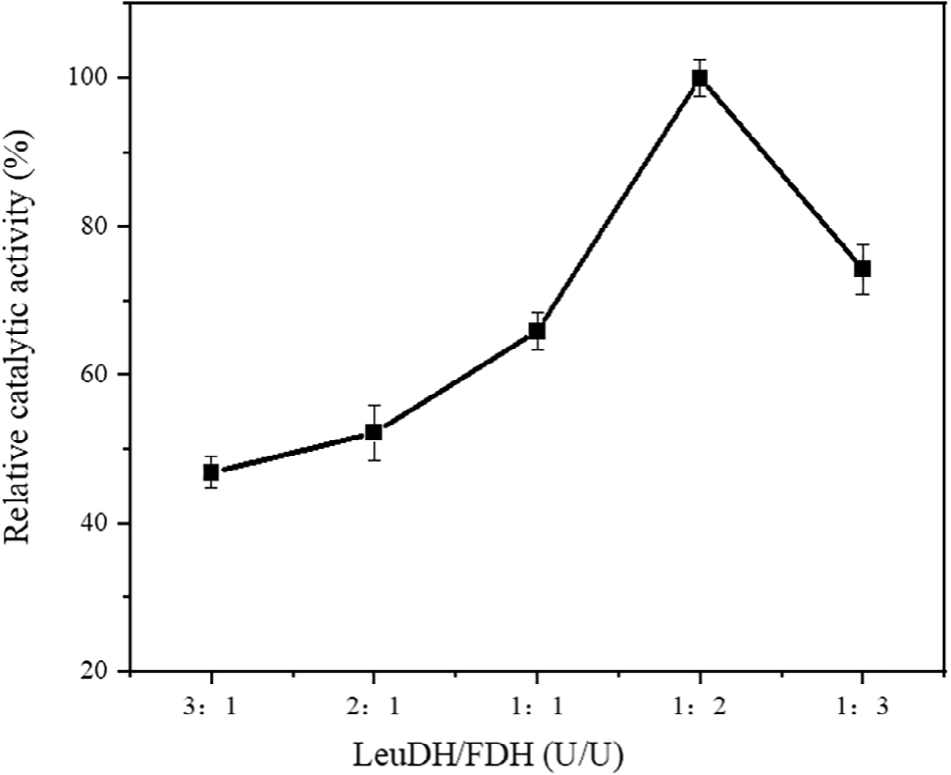
Effect of different enzyme activity ratios on relative catalytic activity. Using CaCl_2_ at a final concentration of 10 mM as the precipitant and glutaraldehyde (pH 7.0) at 0.1% (w/v) as the cross‐linking agent, the activity was determined after cross‐linking at 20°C for 1 h. The relative catalytic activity was expressed as a percentage of the maximum activity under the experimental conditions. Each experiment was conducted in triplicate, and the error bars represent the standard deviation of the mean activity values.

The efficiency of immobilized multi‐enzyme systems in cascade reaction is significantly affected by the catalytic synergy between different enzymes [34]. The proportion of different enzymes determines the amount of each enzyme in the system and subsequently impacts the rate of the catalytic cascade reaction [35]. The effects of different activity ratios of LeuDH and FDH on the relative catalytic activity of combi‐CLEAs were investigated (Figure 4). When the activity ratio of LeuDH and FDH decreased from 3:1 to 1:2, the relative activity of combi‐CLEAs increased from 46.8% to 100%. Conversely, when the ratio of enzyme activity was further decreased to 1:3, the relative activity of the immobilisate declined to 74.2%.

Theoretically, the optimum activity ratio of LeuDH and FDH of 1:1 would ensure that the cofactor NADH produced by the FDH‐catalyzed reaction is fully utilized by LeuDH. Simultaneously, the continuous formation of NAD^+^ in the LeuDH‐catalyzed reaction would serve as a cofactor for FDH, potentially maximizing cofactor regeneration efficiency. However, Figure 4 shows that the relative activity of the immobilisate was highest when the activity ratio of LeuDH and FDH was 1:2. The reason may be that the subunit number and the stability of FDH being less than those of LeuDH, the activity of FDH decreases more during the coprecipitation and cross‐linking process. It is inferred that the combi‐CLEAs, prepared with slightly higher FDH activity than LeuDH, could enhance catalytic efficiency. Then, in the following experiments, the enzyme activity ratio of LeuDH to FDH was at 1:2 for combi‐CLEAs preparation.

#### 3.2.2 Effect of Glutaraldehyde Concentration

**FIGURE 5:**
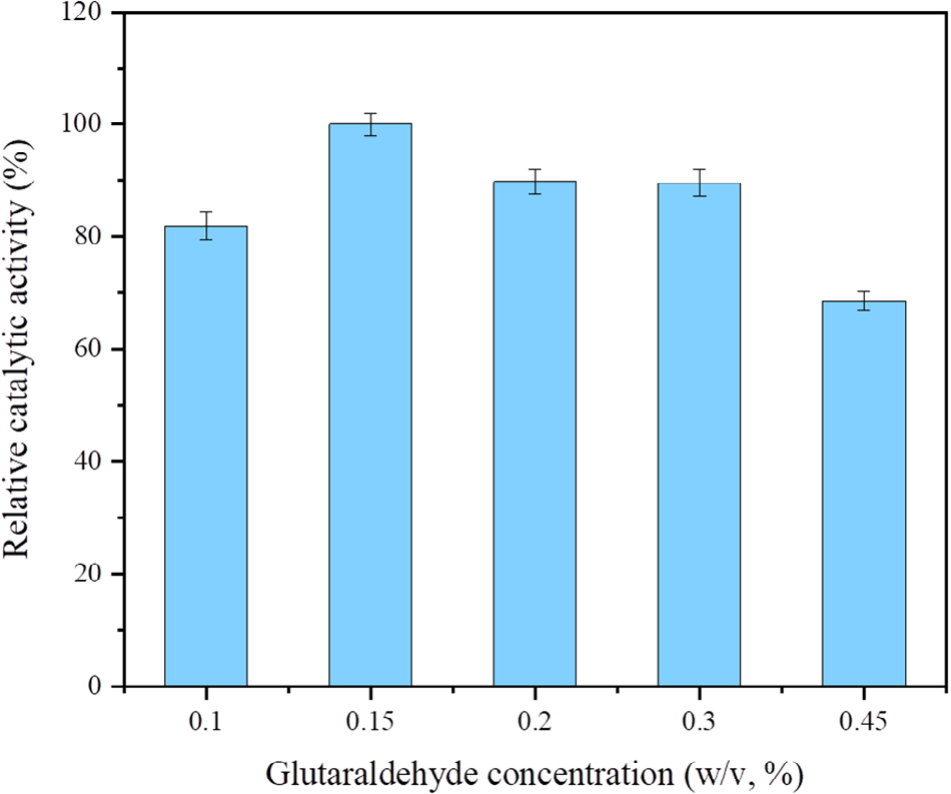
Effect of different concentrations of glutaraldehyde on relative catalytic activity. CaCl_2_, at a final concentration of 10 mM, was used as the precipitant, and LeuDH and FDH were co‐precipitated at an enzyme activity ratio of 1:2. Glutaraldehyde (pH 7.0) served as the cross‐linking agent, and the activity was determined after cross‐linking at 20°C for 1 h. The relative catalytic activity was expressed as a percentage of the maximum activity under the experimental conditions. Each experiment was conducted in triplicate, and the error bars represent the standard deviation of the mean activity values.

The cross‐linking agent plays a key role in the formation of combi‐CLEAs. Glutaraldehyde is a widely used cross‐linking agent because of its compatibility with most enzymes [26]. It can covalently bind with the lysine residues on the enzyme's molecular surface via its two aldehyde groups, effectively “locking” the enzymes and resulting in the formation of insoluble CLEAs. The influence of the glutaraldehyde concentration on the relative catalytic activity of combi‐CLEAs is shown in Figure 5. The results indicate that the optimal catalytic activity is achieved with a 0.15% (w/v) glutaraldehyde concentration. Both insufficient and excessive amounts of cross‐linking agent can lead to reduced enzyme activity. Typically, a lower concentration of cross‐linking agent may result in inadequate cross‐linking and enzyme leaching during the catalytic reaction, whereas an excessively high concentration of glutaraldehyde can cause over‐cross‐linking, leading to a loss of the minimum flexibility required for the enzyme activity [24].

#### 3.2.3 Effect of Cross‐Linking Temperature

**FIGURE 6:**
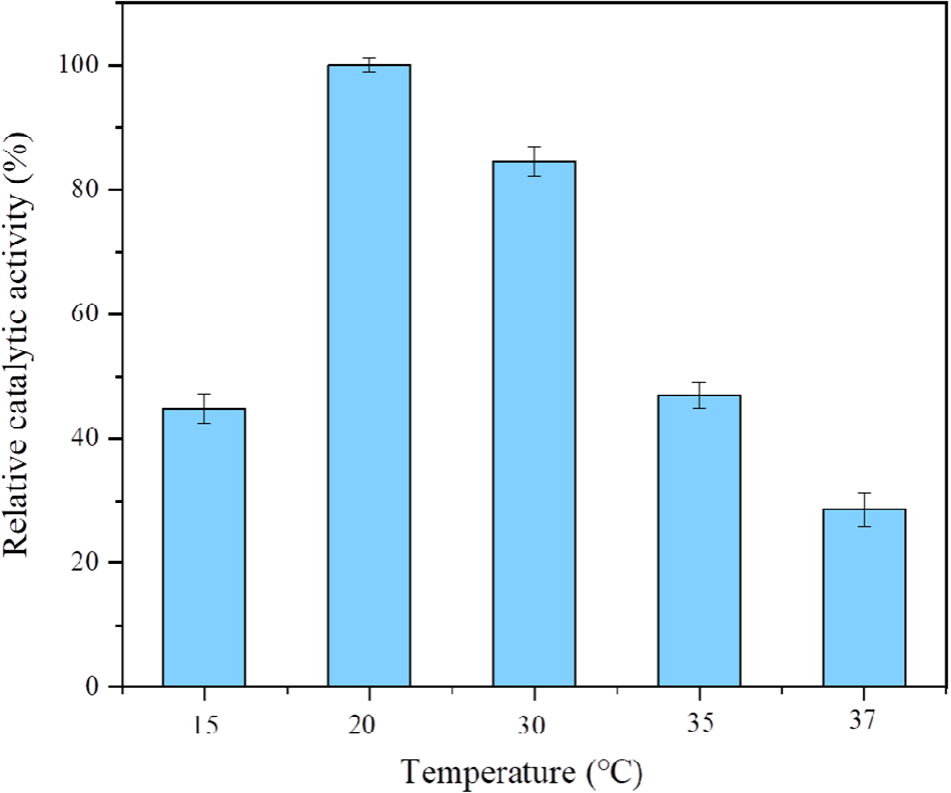
Effect of different cross‐linking temperatures on relative catalytic activity. CaCl_2_, at a final concentration of 10 mM, was used as the precipitant, and glutaraldehyde (pH 7.0) at 0.15% (w/v) was used as the cross‐linking agent. The activity was determined after cross‐linking for 1 h. The relative catalytic activity was expressed as a percentage of the maximum activity under the experimental conditions. Each experiment was conducted in triplicate, and the error bars represent the standard deviation of the mean activity values.

The temperature during the cross‐linking process influences the activity of combi‐CLEAs. It was observed that the relative catalytic activity of the combi‐CLEAs increased by approximately 55% when the temperature was raised from 15°C to 20°C. When the temperature continued to rise, however, its relative activity gradually declined, reaching 28.5% at 37°C (Figure 6). It is speculated that at lower temperatures, the activity of the cross‐linking agent is reduced, leading to insufficient cross‐linking. As the temperature rises, cross‐linking improves because of accelerating thermal motion between the molecules. Nonetheless, excessively high temperatures can adversely affect enzyme activity [36].

#### 3.2.4 Effect of Cross‐Linking pH

**FIGURE 7:**
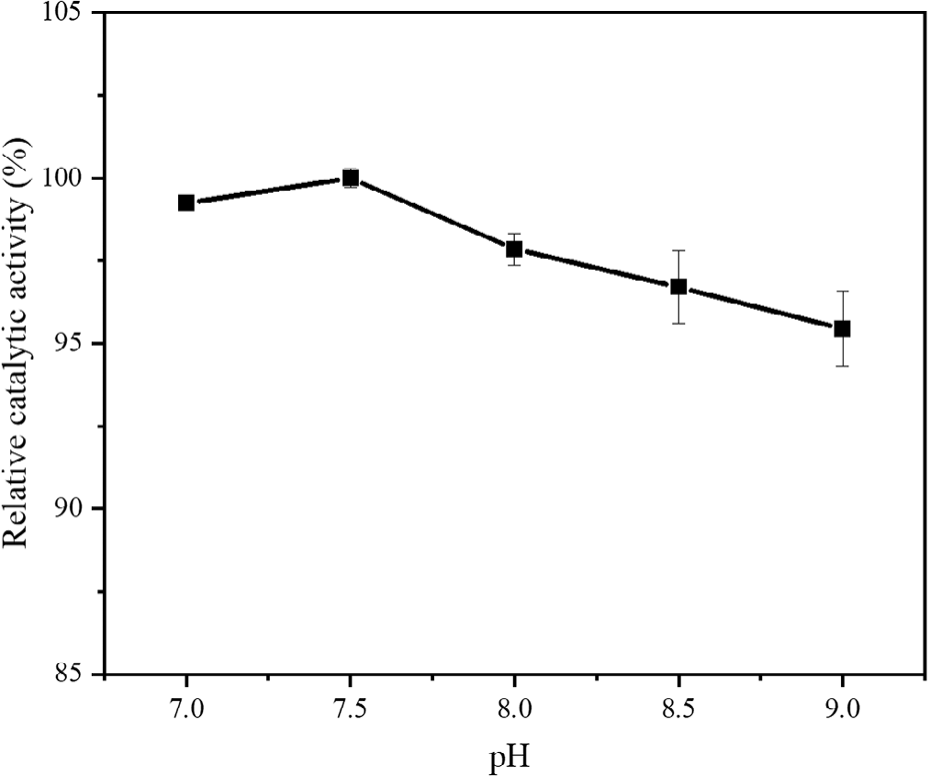
Effect of different cross‐linking pH on relative catalytic activity. CaCl_2_, at a final concentration of 10 mM, was used as the precipitant, and glutaraldehyde at 0.15% (w/v) was used as the cross‐linking agent. The activity was determined after cross‐linking at 20°C for 1 h. The relative catalytic activity was expressed as a percentage of the maximum activity under the experimental conditions. Each experiment was conducted in triplicate, and the error bars represent the standard deviation of the mean activity values.

In the preparation of combi‐CLEAs, pH is another critical parameter. Each enzyme has an optimal pH range within which it functions most efficiently; deviations from this range can lead to conformational changes, causing denaturation and inactivation. The optimal pH values for free LeuDH and free FDH are approximately 9.5 and 7.5, respectively [37, 38]. Furthermore, when using calcium ions as precipitant, the cross‐linking pH may affect the interaction of these ions with LeuDH and FDH [32]. Figure 7 shows that similar relative catalytic activities of combi‐CLEAs were observed at pH ranging from 7.0 to 9.0. Notably, the highest catalytic activity was achieved at pH 7.5. It is implied that within a neutral to weakly alkaline pH range, glutaraldehyde exhibits better reactivity with proteins [39], and both LeuDH and FDH maintain higher activity and stability.

#### 3.2.5 Effect of Cross‐Linking Time

**FIGURE 8:**
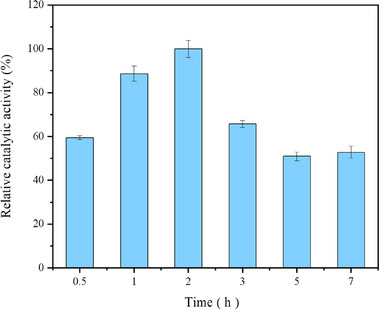
Effect of different cross‐linking times on relative catalytic activity. CaCl_2_, at a final concentration of 10 mM, was used as the precipitant, and LeuDH and FDH were co‐precipitated at an enzyme activity ratio of 1:2. Glutaraldehyde (pH 7.5) at 0.15% (w/v) was used as the cross‐linking agent. The activity was determined after cross‐linking at 20°C for different durations. The relative catalytic activity was expressed as a percentage of the maximum activity under the experimental conditions. Each experiment was conducted in triplicate, and the error bars represent the standard deviation of the mean activity values.

Determining the optimal cross‐linking time is a balance between achieving effective cross‐linking and maintaining enzyme activity. Cross‐linking durations ranging from 0.5 to 7 h were investigated. The results revealed a trend of initial increase followed by a decrease in relative catalytic activity (Figure 8). The relative catalytic activity of the combi‐CLEAs increased with cross‐linking time, peaking at 2 h, after which it declined with extended cross‐linking times. This suggests that enzyme aggregates do not achieve sufficient cross‐linking if the duration is less than 2 h. However, extended cross‐linking times beyond 2 h result in a decrease in enzyme activity, potentially due to over‐cross‐linking, where the free aldehyde groups of glutaraldehyde fully react with enzymes, causing damage [36, 40].

### 3.3 Characterization of Combi‐CLEAs

#### 3.3.1 Kinetic Parameters of the Immobilized Catalysts

The immobilization of LeuDH and FDH in combi‐CLEAs resulted in an increase in the apparent *K*_m_ value from 35.21 to 42.11 mM, indicating a slightly reduced affinity of the immobilized enzymes for the substrate. Additionally, the apparent *V*_max_ value decreased from 12.31 to 10.64 µmol/min upon immobilization (Table 3). This reduction in *V*_max_ may be attributed to the rigid supramolecular structure formed during aggregation, which potentially restricts enzyme activity.

**TABLE 3:** Kinetic parameters of free enzyme mixture of LeuDH and FDH and combi‐CLEAs.

Enzyme preparation	*V*_max_ (µmol min^−1^)	*K*_m_ (mM)
Free enzymes	12.31	35.21
Combi‐CLEAs	10.64	42.11

#### 3.3.2 Optimal Reaction pH and Temperature

**FIGURE 9:**
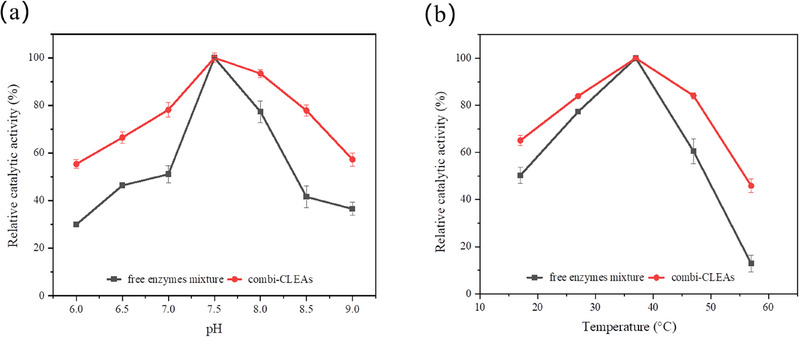
The effect of pH (a) and temperature (b) on the relative catalytic activity of combi‐CLEAs and free enzyme mixture during the catalysis process. The relative catalytic activity was expressed as a percentage of the maximum activity of combi‐CLEAs and free enzyme mixture under the experimental conditions. The experiments were conducted in triplicate, and the error bars represent the standard deviation of the mean activity values.

In this study, the free enzyme mixture of LeuDH and FDH was explored collectively rather than as separated entities. The effects of reaction pH and temperature on the relative catalytic activity of both combi‐CLEAs and the free enzymes mixture are shown in Figure 9. It is evident that for both systems, optimal catalytic activities were achieved at pH 7.5 using a phosphate buffer. However, combi‐CLEAs demonstrated superior relative activity across other pH values (Figure 9a). This result is explained by the optimal pH levels of LeuDH and FDH, which are around 9.5 and 7.5, respectively, with FDH being less stable. At lower pH levels, the activities of both enzymes are compromised. On the contrary, at higher pH levels, the activity of FDH is markedly suppressed. In addition, cross‐linking enhances enzyme rigidity, shielding its active site. In other words, the stability of enzyme molecules in combi‐CLEAs under the catalysis of pH extremes is improved.

Figure 9b illustrates that the optimal catalytic temperature for both combi‐CLEAs and free enzymes mixture was 37°C. However, combi‐CLEAs exhibited a notable increase in relative catalytic activity at higher catalytic temperatures. When catalyzed at 57°C, the relative activity of the free enzyme mixture was 13%, whereas that of the combi‐CLEAs was 45%. This enhancement is likely due to the covalent bonding between the cross‐linking agent glutaraldehyde and the protein molecules, which increases enzyme rigidity and prevents conformational changes. Thus, the heat resistance of enzymes within combi‐CLEAs is improved during the catalytic process [27].

#### 3.3.3 Thermal Stability

**FIGURE 10:**
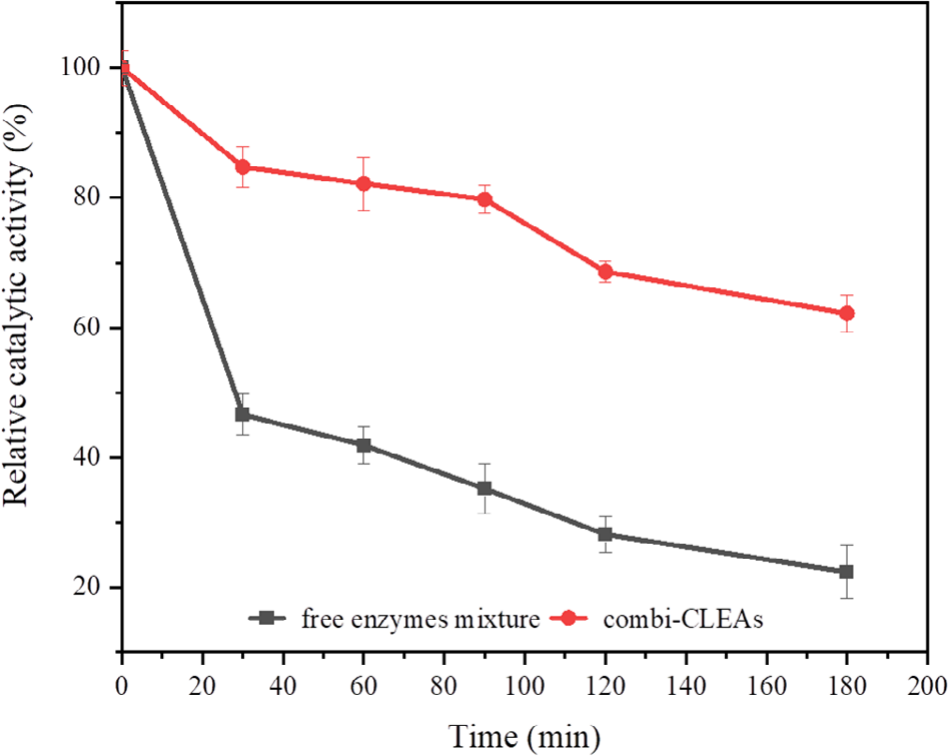
Thermal stability of free enzyme mixture and combi‐CLEAs. The maximum activity of the free enzyme mixture and combi‐CLEAs was set as 100%. The experiments were conducted in triplicate, and the error bars represent the standard deviation of the mean activity values.

For industrial applications, the thermal stability of immobilized preparations is crucial. The thermal stabilities of both combi‐CLEAs and free enzymes mixture were examined at 45°C over different time intervals (Figure 10). The results displayed that the stability of combi‐CLEAs was increased significantly. After 180 min of incubation, the activity of the free enzymes mixture dropped below 30%, whereas combi‐CLEAs retained more than 60% of its initial activity. This enhancement in stability can be due to inter‐ and intramolecular covalent cross‐linking [27] and the orderly arrangement of enzyme molecules during the cross‐linking process [24]. Furthermore, these results also imply that co‐immobilization of enzymes as combi‐CLEAs would endow the immobilized enzymes with more pronounced protection against thermal denaturation, requiring more energy to disrupt the active conformation than that of the free enzymes mixture [41].

#### 3.3.4 pH Stability

**FIGURE 11:**
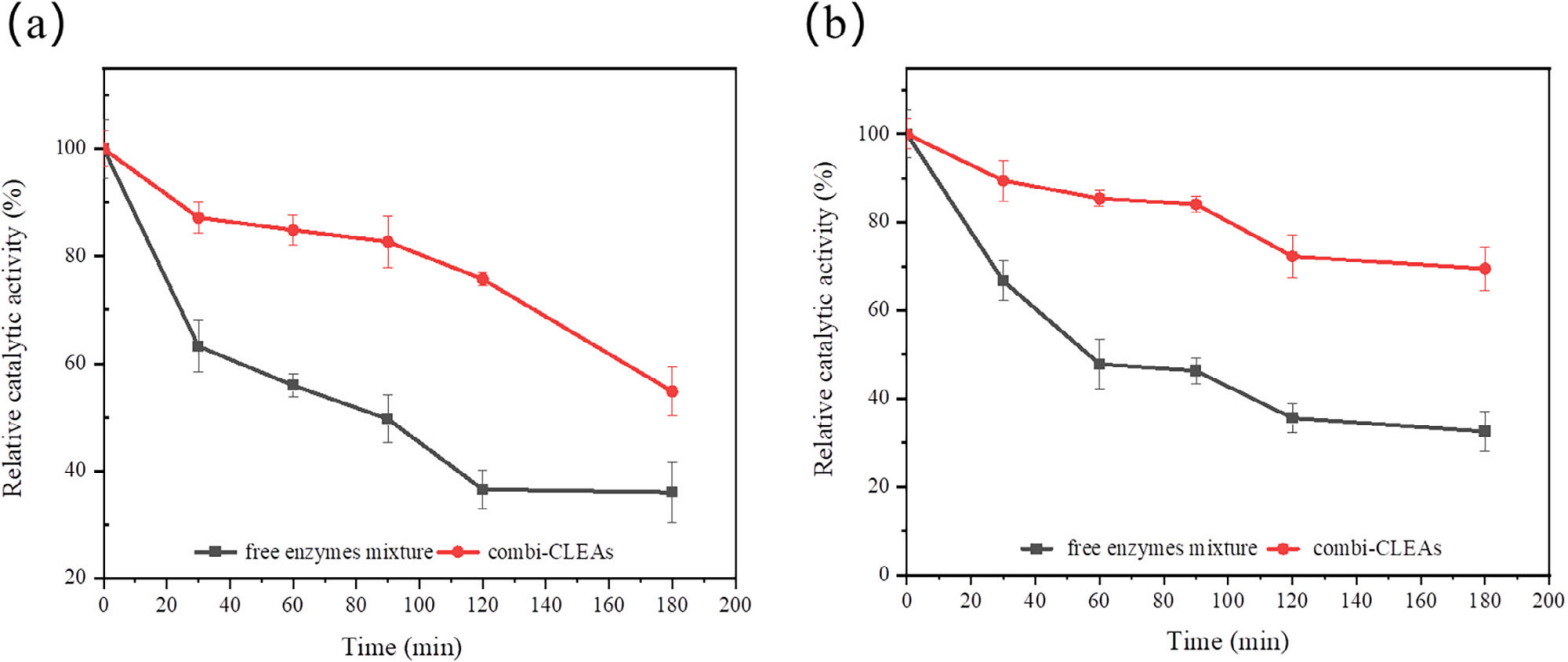
Effect of pH on free enzyme mixture and combi‐CLEAs at pH 6.0 (a) and pH 10.0 (b). The maximum activity of the free enzyme mixture and combi‐CLEAs was set as 100%. The experiments were conducted in triplicate, and the error bars represent the standard deviation of the mean activity values.

pH is another key factor that affects catalytic stability. Enzymes are often immobilized to improve their tolerance to changes in the pH of reaction system. The pH tolerance of combi‐CLEAs and free enzymes mixture at pH 6.0 (Figure 11a) and pH 10.0 (Figure 11b) was investigated in the absence of substrate. The activity of the free enzymes mixture decreased considerably in both acidic and alkaline conditions over time, whereas the combi‐CLEAs showed no significant decline in activity. After incubation at pH 6.0 and pH 10.0 for 180 min, the relative catalytic activity of the free enzymes mixture decreased to 36.0% and 32.6% of its initial activity, respectively. In contrast, combi‐CLEAs maintained 54.8% and 69.4% of its initial activity after 180 min of incubation at pH 6.0 and pH 10.0, respectively. Compared to the free enzymes mixture, this increase in activity of combi‐CLEAs might be due to the cross‐linking process, which greatly protected the enzyme's active conformation [36].

#### 3.3.5 Reusability of the Combi‐CLEAs

**FIGURE 12:**
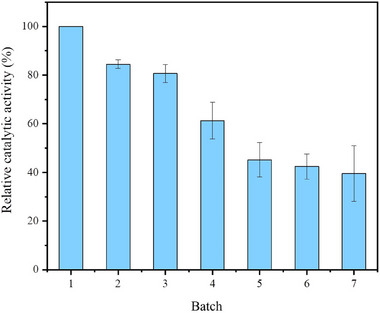
Reusability of the combi‐CLEAs. The activity of the combi‐CLEAs in the first cycle was set as a 100% activity control. The experiments were conducted in triplicate, and the error bars represent the standard deviation of the mean activity values.

A primary advantage of immobilized enzymes over free enzyme is their potential for reuse, which is crucial for cost‐effective applications [42, 43]. To assess the reusability of combi‐CLEAs, the enzyme activity was determined over multiple cycles. As shown in Figure 12, a gradual decline in relative catalytic activity decay was observed over 15‐min reaction intervals. Compared to 100% relative activity of the first cycle, the combi‐CLEAs maintained 40% of its initial activity after seven recycling cycles. It is well known that the continuous stirring during reaction, followed by centrifugation and washing, can lead to the loss of enzyme preparations, thereby reducing activity. Moreover, the decline in activity with an increasing number of recycles may be attributed to enzyme leaching from the combi‐CLEAs, where weaker bonds between enzyme molecules exist, and enzyme inactivation during the reaction process [27].

#### 3.3.6 Scanning Electron Microscopy

**FIGURE 13:**
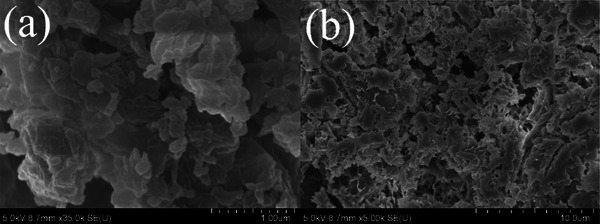
Scanning electron microscopy images of (a) combi‐CLEAs at 35,000× magnification and (b) combi‐CLEAs at 5000× magnification.

The morphology of combi‐CLEAs was analyzed using scanning electron microscopy (SEM), and the results are presented in Figure 13. The SEM images revealed that the combi‐CLEAs exhibited a thick, irregularly shaped structure, indicative of microparticles aggregating into units with diameters ranging from 1 to 10 µm. Thus, the combi‐CLEAs did not exhibit a uniform shape.

#### 3.3.7 Confocal Fluorescence Microscopy

**FIGURE 14:**
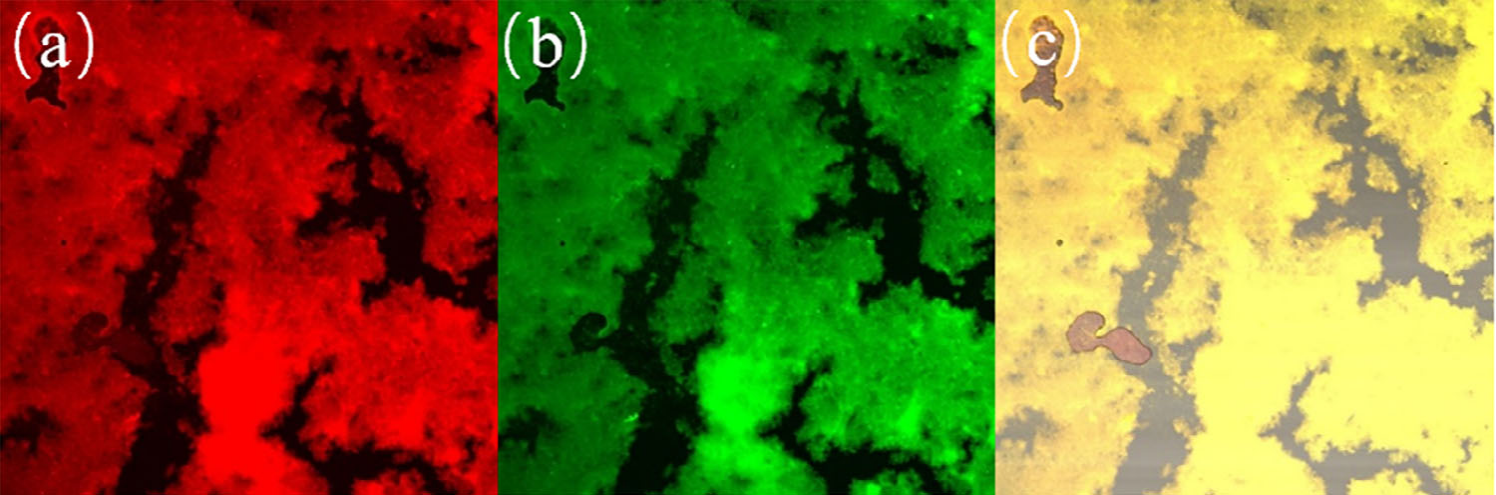
Confocal laser scanning microscopy images of combi‐CLEAs with fluorescence labeling at different excitation wavelengths. The red (a) and green (b) fluorescence is at 495 and 570 nm, respectively, in single fluorescence detection channels, and the yellow fluorescence (c) is at 495 and 570 nm in dual fluorescence detection channels.

To further verify the co‐immobilization of LeuDH and FDH within the combi‐CLEAs structure, LeuDH was labeled with FITC and FDH was labeled with RhoB. These labeled enzymes were used to prepare combi‐CLEAs, and their fluorescence characteristics were examined using a laser confocal microscope (Figure 14).

Under excitation with different wavelengths, the combi‐CLEAs exhibited distinct fluorescence signals. Specifically, green fluorescence corresponding to FITC‐labeled LeuDH and red fluorescence corresponding to RhoB‐labeled FDH were observed. Additionally, regions of yellow fluorescence were detected, resulting from the merging of green and red fluorescence. This colocalization of green and red fluorescence within the same region confirms that both LeuDH and FDH were successfully co‐immobilized in the combi‐CLEAs. These findings provide strong evidence for the effective integration of the two enzymes within the combi‐CLEAs structure, suggesting that the immobilization process facilitated close spatial proximity between LeuDH and FDH.

## 4 Concluding Remarks

This study demonstrates the feasibility of using a low concentration of calcium ions as the precipitant for the preparation of combi‐CLEAs of LeuDH and FDH for the in situ regeneration of NADH cofactor. This approach is simple, cost‐effective, and environmentally friendly, making it particularly suitable for preparing combi‐CLEAs from the recombinant His‐tagged enzymes. The optimal parameters for preparing combi‐CLEAs were established: Ca^2+^ concentration of 10 mM, an activity ratio of LeuDH and FDH of 1:2, glutaraldehyde at 0.15% (w/v), cross‐linking temperature of 20°C, cross‐linking pH of 7.5, and cross‐linking time of 2 h. The optimal catalytic temperature and pH value of combi‐CLEAs were 37°C and 7.5, respectively. The combi‐CLEAs exhibited superior thermal and pH stability compared to the free enzyme mixture and demonstrated improved operational stability, retaining 40% of its initial activity after seven cycles of reuse. These findings suggest that combi‐CLEAs of LeuDH and FDH are highly promising as a carrier‐free immobilized biocatalyst for the industrial production of 2‐aminobutyric acid.

## Conflicts of Interest

The authors declare no conflicts of interest.

## Supporting information



Supporting Information
