# Supplementary material for: A Cofactor Regeneration System for 2‐Aminobutyric Acid Production Based on Combined Cross‐Linked Enzyme Aggregates: Utilizing His‐Tagged Enzymes With Low‐Concentration Calcium Ions as Precipitant
Source: Eng Life Sci. 2025 Feb 28;25(3):e70013. doi: 10.1002/elsc.70013 (PMC11870830; doi:10.1002/elsc.70013)
Supplement: Supplementary file 1 — Supporting Information [file ELSC-25-e70013-s001.docx]

**Supplementary Information**

**A Cofactor Regeneration System for 2-Aminobutyric Acid Production Based on Combined Cross-Linked Enzyme Aggregates: Utilizing His-Tagged Enzymes with Low-Concentration Calcium Ion as Precipitant**

Jingran Liu ^1,2,3 ǂ^, Ren Li ^1,2,3 ǂ^, Hongxu Sun ^2^, Qiwei Chen ^2^, Haiyan Song ^1,2,3^, Hui Peng ^1,2,3^, Yanhong Chang ^1,3*^, Hui Luo ^2*^

^1^ Department of Environmental Science and Engineering, University of Science and Technology Beijing, Beijing 100083, China

^2^ Department of Biological Science and Engineering, University of Science and Technology Beijing, Beijing 100083, China

^3^ Beijing Key Laboratory of Resource-oriented Treatment of Industrial Pollutants, Beijing 100083, China

Correspondence: Y. Chang (yhchang@ustb.edu.cn); H. Luo (luohui@ustb.edu.cn)

^ǂ^ Co-first authors. They contributed equally to this article.

**The nucleic acid sequences of LeuDH**

1 ATGGGCAGCA GCCATCATCA TCATCATCAC AGCAGCGGCC TGGTGCCGCG CGGCAGCCAT

61 ATGGCTAGCA TGACTGGTGG ACAGCAAATG GGTCGCGGAT CCATGACCCT GGAAATCTTC

121 GAATACCTGG AAAAATACGA CTACGAACAG GTTGTTTTCT GCCAGGACAA AGAATCTGGT

181 CTGAAAGCTA TCATCGCTAT CCACGACACC ACCCTGGGTC CGGCTCTGGG TGGTACCCGT

241 ATGTGGACCT ACGACTCTGA AGAAGCTGCT ATCGAAGACG CTCTGCGTCT GGCTAAAGGT

301 ATGACCTACA AAAACGCTGC TGCTGGTCTG AACCTGGGTG GTGCTAAAAC CGTTATCATC

361 GGTGACCCGC GTAAAGACAA ATCTGAAGCT ATGTTCCGTG CTCTGGGTCG TTACATCCAG

421 GGTCTGAACG GTCGTTACAT CACCGCTGAA GACGTTGGTA CCACCGTTGA CGACATGGAC

481 ATCATCCACG AAGAAACCGA CTTCGTTACC GGTATCTCTC CGTCTTTCGG TTCTTCTGGT

541 AACCCGTCTC CGGTTACCGC GTATGGTGTA TACCGGGGTA TGAAAGCTGC TGCTAAGGAG

601 GCGTTCGGTA CCGACAATCT GGAAGGTAAA GTTATCGCTG TTCAGGGTGT TGGTAACGTT

661 GCTTACCACC TGTGCAAACA CCTGCACGCT GAAGGTGCTA AACTGATCGT TACCGACATC

721 AACAAAGAAG CTGTTCAGCG TGCTGTTGAA GAATTCGGTG CTTCTGCTGT TGAACCGAAC

781 GAAATCTACG GTGTTGAATG CGACATCTAC GCTCCGTGCG CTCTGGGTGC TACCGTTAAC

841 GACGAAACCA TCCCGCAGCT GAAAGCTAAA GTTATCGCTG GTTCTGCTAA CAACCAGCTG

901 AAAGAAGACC GTCACGGTGA CATCATCCAC GAAATGGGTA TCGTTTACGC TCCGGACTAC

961 GTTATCAACG CTGGTGGTGT TATCAACGTT GCTGACGAAC TGTACGGTTA CAACCGTGAA

1021 CGTGCTCTGA AACGTGTTGA ATCTATCTAC GACACCATCG CTAAAGTTAT CGAAATCTCT

1081 AAACGTGACG GTATCGCTAC CTACGTTGCT GCTGACCGTC TGGCTGAAGA ACGTATCGCT

1141 TCTCTGAAAA ACTCTCGTTC TACCTACCTG CGTAACGGTC ATGATATTAT TTCTCGTCGT

1201 TAA

**The nucleic acid sequences of FDH**

1 ATGGGCAGCA GCCATCATCA TCATCATCAC AGCAGCGGCC TGGTGCCGCG CGGCAGCCAT

61 ATGGCTAGCA TGACTGGTGG ACAGCAAATG GGTCGCGGAT CCATGAAAAT CGTTCTGGTT

121 CTGTACGACG CTGGTAAACA CGCTGCTGAC GAAGAAAAAC TGTACGGTTG CACCGAAAAC

181 AAACTGGGTA TCGCTAACTG GCTGAAAGAC CAGGGTCACG AACTGATCAC CACCTCTGAC

241 AAAGAAGGTG GTAACTCTGT TCTGGACCAG CACATCCCGG ACGCTGACAT CATCATCACC

301 ACCCCGTTCC ACCCGGCTTA CATCACCAAA GAACGTATCG ACAAAGCTAA AAAACTGAAA

361 CTGGTTGTTG TTGCTGGTGT TGGTTCTGAC CACATCGACC TGGACTACAT CAACCAGACC

421 GGTAAAAAAA TCTCTGTTCT GGAAGTTACC GGTTCTAACG TTGTTTCTGT TGCTGAACAC

481 GTTGTTATGA CCATGCTGGT TCTGGTTCGT AACTTCGTTC CGGCTCACGA ACAGATCATC

541 AACCACGACT GGGAAGTTGC TGCTATCGCT AAAGACGCTT ACGACATCGA AGGTAAAACC

601 ATCGCTACCA TCGGTGCTGG TCGTATCGGT TACCGTGTTC TGGAACGTCT GGTTCCGTTC

661 AACCCGAAAG AACTGCTGTA CTACGACTAC CAGGCTCTGC CGAAAGACGC TGAAGAAAAA

721 GTTGGTGCTC GTCGTGTTGA AAACATCGAA GAACTGGTTG CTCAGGCTGA CATCGTTACC

781 GTTAACGCTC CGCTGCACGC TGGTACCAAA GGTCTGATCA ACAAAGAACT GCTGTCTAAA

841 TTCAAAAAAG GTGCTTGGCT GGTTAACACC GCTCGTGGTG CTATCTGCGT TGCTGAAGAC

901 GTTGCTGCTG CTCTGGAATC TGGTCAGCTG CGTGGTTATG GCGGTGACGT GTGGTTCCCC

961 CAGCCGGCTC CGAAAGACCA CCCGTGGCGT GACATGCGTA ACAAATACGG TGCTGGTAAC

1021 GCTATGACCC CGCACTACTC TGGTACCACC CTGGACGCTC AGACCCGTTA CGCTCAGGGT

1081 ACCGTTAACA TCCTGGAATC TTTCTTCACC GGTAAATTCG ACTACCGTCC GCAGGACATC

1141 ATCCTGCTGA ACGGTGAATA CGTTACCAAG GCTTACGGTA AACATGATAA AAAATAA
